# Phase I Trial of Autologous RNA-electroporated cMET-directed CAR T Cells Administered Intravenously in Patients with Melanoma and Breast Carcinoma

**DOI:** 10.1158/2767-9764.CRC-22-0486

**Published:** 2023-05-09

**Authors:** Payal D. Shah, Alexander C. Huang, Xiaowei Xu, Robert Orlowski, Ravi K. Amaravadi, Lynn M. Schuchter, Paul Zhang, Julia Tchou, Tina Matlawski, Amanda Cervini, Joanne Shea, Joan Gilmore, Lester Lledo, Karen Dengel, Amy Marshall, E. John Wherry, Gerald P. Linette, Andrea Brennan, Vanessa Gonzalez, Irina Kulikovskaya, Simon F. Lacey, Gabriela Plesa, Carl H. June, Robert H. Vonderheide, Tara C. Mitchell

**Affiliations:** 1 Perelman School of Medicine at the University of Pennsylvania, Philadelphia, Pennsylvania.; 2 Abramson Cancer Center, University of Pennsylvania, Philadelphia, Pennsylvania.; 3 Center for Cellular Immunotherapies, Perelman School of Medicine at the University of Pennsylvania, Philadelphia, Pennsylvania.; 4 Department of Systems Pharmacology and Translational Therapeutics, Institute of Immunology, Perelman School of Medicine at the University of Pennsylvania, Philadelphia, Pennsylvania.

## Abstract

**Purpose:**

Treatments are limited for metastatic melanoma and metastatic triple-negative breast cancer (mTNBC). This pilot phase I trial (NCT03060356) examined the safety and feasibility of intravenous RNA-electroporated chimeric antigen receptor (CAR) T cells targeting the cell-surface antigen cMET.

**Experimental Design:**

Metastatic melanoma or mTNBC subjects had at least 30% tumor expression of cMET, measurable disease and progression on prior therapy. Patients received up to six infusions (1 × 10e8 T cells/dose) of CAR T cells without lymphodepleting chemotherapy. Forty-eight percent of prescreened subjects met the cMET expression threshold. Seven (3 metastatic melanoma, 4 mTNBC) were treated.

**Results:**

Mean age was 50 years (35–64); median Eastern Cooperative Oncology Group 0 (0–1); median prior lines of chemotherapy/immunotherapy were 4/0 for TNBC and 1/3 for melanoma subjects. Six patients experienced grade 1 or 2 toxicity. Toxicities in at least 1 patient included anemia, fatigue, and malaise. One subject had grade 1 cytokine release syndrome. No grade 3 or higher toxicity, neurotoxicity, or treatment discontinuation occurred. Best response was stable disease in 4 and disease progression in 3 subjects. mRNA signals corresponding to CAR T cells were detected by RT-PCR in all patients’ blood including in 3 subjects on day +1 (no infusion administered on this day). Five subjects underwent postinfusion biopsy with no CAR T-cell signals seen in tumor. Three subjects had paired tumor tissue; IHC showed increases in CD8 and CD3 and decreases in pS6 and Ki67.

**Conclusions:**

Intravenous administration of RNA-electroporated cMET-directed CAR T cells is safe and feasible.

**Significance:**

Data evaluating CAR T therapy in patients with solid tumors are limited. This pilot clinical trial demonstrates that intravenous cMET-directed CAR T-cell therapy is safe and feasible in patients with metastatic melanoma and metastatic breast cancer, supporting the continued evaluation of cellular therapy for patients with these malignancies.

## Introduction

Despite significant therapeutic advances in metastatic melanoma and metastatic triple-negative breast cancer (mTNBC), these diseases remain formidable contributors of cancer-related mortality and morbidity, warranting the exploration of novel approaches. For patients with metastatic melanoma, molecularly targeted therapies and immune checkpoint inhibitors have improved survival (1–5) but many patients have treatment-resistant disease, and long-term survival remains poor for these patients. The treatment of mTNBC is limited largely to cytotoxic chemotherapy, with immune checkpoint inhibitors (6, 7) and antibody–drug conjugates (8) more recently approved but effective in a minority of patients. Chimeric antigen receptor (CAR) T-cell therapy has demonstrated efficacy in hematologic malignancies (9–12), resulting in approvals by the FDA (13–15). CAR T cells exert antitumor effects by pairing the MHC-independent tumor-recognition capabilities of mAbs with the cytotoxicity of effector T cells.

The *MET* oncogene encodes for hepatocyte growth factor receptor, a cell surface protein tyrosine kinase physiologically expressed on the surface of epithelial cells within the liver, pancreas, prostate, kidney, muscle, and bone marrow. *MET* is also expressed in multiple solid tumors and is widely implicated in tumor cell proliferation, invasion, and metastasis (16–29). High levels of MET correlate with poor prognosis in breast cancer (22, 30–34), and therapeutic targeting of cMET has been of interest in breast, melanoma (35, 36), and other cancers. MET is enriched in basal-like, TNBCs (24, 37) as well as in metastatic melanoma (28) and was therefore a rational target antigen for CAR T therapeutic approaches.

A prior study evaluated intratumoral administration of mRNA-transfected cMET CAR T cells in patients with metastatic breast cancer (37). Injections were well tolerated, and no hepatic toxicity was seen despite 2 subjects having detectable CAR mRNA transgene in the peripheral blood. Histologic examination of posttreatment tumor demonstrated extensive tumor necrosis, loss of cMET immunoreactivity, and cellular debris at the injection site with macrophages at the leading edges and within necrotic zones, suggesting an inflammatory response evoked by treatment. We thus conducted a pilot clinical trial examining systemic administration of RNA-electroporated cMET CAR T cells without lymphodepleting chemotherapy in subjects with metastatic melanoma and mTNBC (NCT03060356). In the current study, mRNA cMET-directed CAR T cells were studied because (i) electroporation of mRNA-encoded CAR T cells ensured transient CAR expression as a safety feature and (ii) data from the study of mRNA mesothelin-directed CAR T cells in patients with mesothelioma and pancreas cancer suggested the ability of systemically infused mRNA CAR T cells to traffick into the tumor microenvironment and demonstrate antitumor activity (38, 39). To the authors’ knowledge, this study represents the first published clinical trial of systemic CAR T administration in patients with melanoma and breast cancer.

## Materials and Methods

### Study Participants

Subjects were identified through the medical oncology clinical practices of the Abramson Cancer Center at the University of Pennsylvania (Philadelphia, PA), affiliated hospitals, and through referrals from outside hospitals and physicians. Eligible patients were adults ages ≥18 years with unresectable or mTNBC, as defined by lack of expression of the estrogen and progesterone receptors and lack of HER2 overexpression, or unresectable or metastatic melanoma. Tumors were required to have cMET expression in at least 30% of tumor cells (felt to represent at least moderate expression) based on IHC analysis at the University of Pennsylvania (Philadelphia, PA) performed on a primary or metastatic tumor sample either from screening biopsy or archival slides. Subjects were required to have an Eastern Cooperative Oncology Group (ECOG) performance status of 0 or 1, measurable disease as per RECIST version 1.1 in addition to a second site of disease accessible for biopsy or surgical resection, and disease progression on at least one prior therapy for advanced stage disease. Subjects were not required to have disease progression at the time of study enrollment. Adequate hematologic, renal, hepatic, and cardiac function was required and was defined by a serum creatinine ≤1.5 times upper limit of normal; total bilirubin, aspartate aminotransferase, and alanine aminotransferase ≤2 times upper limit of normal; and cardiac ejection fraction of ≥40% by echocardiogram. If prior immunotherapy was received, the last administered treatment was required to be received at least 2 weeks prior to study enrollment; prior genetically modified T cells were not permitted. Individuals with human immunodeficiency virus, active hepatitis B or C, or comorbidities felt to interfere with protocol compliance or interpretation of study results were excluded from study participation.

The clinical trial protocol was approved by the Institutional Review Board at the University of Pennsylvania (Philadelphia, PA). All participants provided written informed consent prior to the conduct of any study-related procedures. The study was conducted in accordance with the FDA regulations, the International Conference on Harmonization Guidelines for Good Clinical Practice, and the Declaration of Helsinki.

### Study Design and Treatment Plan

This trial was an open-label, phase I study of RNA-electroporated CAR T cells directed against cMET in adults with cMET-expressing mTNBC and metastatic melanoma (ClinicalTrials.gov: NCT01837602). The primary objective was to determine the safety and feasibility of intravenously administered RNA-electroporated CAR T cells directed against cMET. Secondary objectives were to estimate preliminary activity of the investigational therapy and to assess persistence and trafficking of the CAR T-cell product.

Subjects were screened for eligibility prior to undergoing a large-volume leukapheresis for collection of peripheral blood mononuclear cells used for CAR T-cell manufacturing. Screening included IHC assessment of baseline cMET expression in tumor sample semiquantitatively by a laboratory developed test using an *in vitro* diagnostic rabbit anti-human cMET antibody (Confirm, clone Sp44, Ventana) developed and performed by a Clinical Laboratory Improvement Amendments-certified IHC laboratory at the Hospital of the University of Pennsylvania (HUP), Department of Pathology and Laboratory Medicine. Screening cMET immunostain was evaluated by a single pathologist (P. Zhang). Autologous T cells were engineered to express a cMET CAR by electroporation of *in vitro* transcribed mRNA (40). The cMET CAR contained an extracellular single-chain antibody variable fragment (scFv) with MET specificity using sequences from a previously developed 5D5 antibody (41). The CAR moiety contained intracellular signaling domains comprised of the TCR-ζ chain and CD137 (4-1BB) developed at our institution (42). Manufacturing and release testing was performed by the CVPF at the University of Pennsylvania (Philadelphia, PA). Cell release was dependent upon meeting FDA-specified release criteria for infused cells.

**FIGURE 1:**
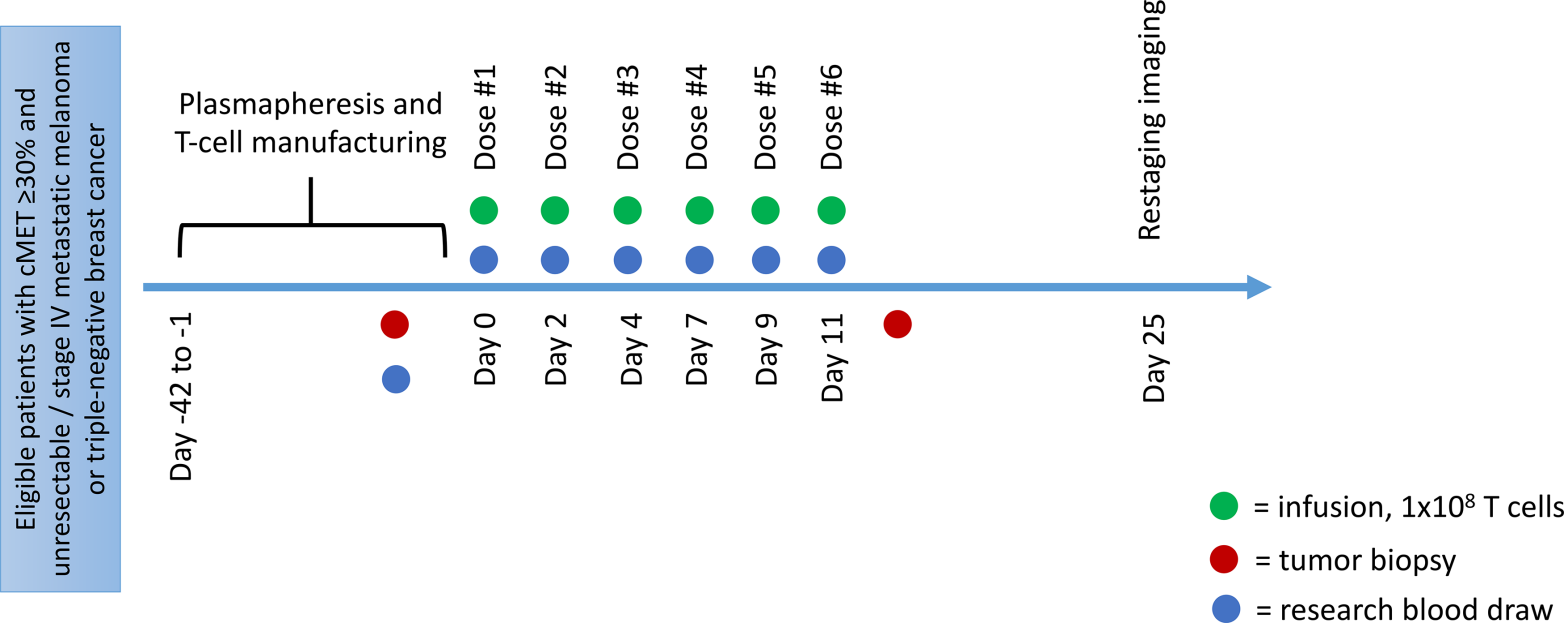
Study procedures. Participant study procedures including cell collection/manufacturing timeline as well as infusion schedule from eligibility confirmation onward.

Study schema is shown in Fig. 1. Study participants received doses of 1 × 10^8^ T cells modified with RNA anti-cMET CAR administered by intravenous infusion, with planned administration of six doses over a period of 14 days. The dose of 1 × 10e8 T cells/dose was the standard dose of cells used the University of Pennsylvania (Philadelphia, PA) at the time in other studies (38). The following considerations informed dosing schedule: (i) given the limited persistence of RNA-based CARs (38), frequent infusions were felt to promote a steady level of RNA CARs in the circulation as a source for tumor uptake; (ii) intermittent dosing was felt to be more feasible and less burdensome to patients than daily infusions and had been successfully employed previously (39); (iii) prior experience (43) suggested a risk of IgE-mediated anaphylactic events with infusions separated over a greater number of days and occurring over a greater number of elapsed days, and practice at the University of Pennsylvania (Philadelphia, PA) was thus to separate infusions by less than 10 days and complete all infusions within 21 days. Antecedent lymphodepleting chemotherapy was not administered as this was a pilot study of CAR T-cell administration. If subjects’ manufactured product did not meet the target quantity of cells for six doses, the number of doses was planned to be reduced such that the target cell number per dose was met. Therapy was administered on an outpatient basis. Subjects were permitted to receive antineoplastic therapy between the time of leukapheresis and CAR T-cell infusion during the period of cell manufacturing; however, no systemic therapy was permitted within 2 weeks prior to RNA CAR T-cell infusion. Following cell infusion, subjects did not receive active treatment for their cancer until after day +25 disease assessment unless medically indicated for clinical disease progression.

### Study Procedures

Safety assessments included evaluation of adverse events as per the NCI Common Terminology Criteria for Adverse Events version 4.03. Adverse event assessment was performed at the following timepoints: at each infusion; on day +1 following the first infusion; on the date of tumor resection or biopsy which was on day 11 or within 3 days of last CAR T infusion; at end of treatment; at each monthly follow-up visit until 4 months after the first infusion or until alternative therapy was initiated, whichever occurred earlier. A treatment-limiting toxicity (TLT) was defined as any grade 3 non-hematologic toxicity which is new, develops following dosing within 14 days of final T-cell administration, and is at least possibly related to CAR T cells. TLTs did not include adverse events that resolved to grade 1–2 within 7 days, electrolyte disturbances, nausea, vomiting, diarrhea, or fatigue. TLTs did include grade 2 or higher autoimmune reactions and grade 3 or higher allergic reactions.

Response assessments included CT or other cross-sectional imaging assessed using the RECIST version 1.1 (RECIST v1.1; ref. 44). Response as measured using the immune-related response criteria was permissible if feasible (45). Imaging was performed at baseline and on day +25. For subjects without disease progression at day 25, imaging was performed at 4 months for patients with melanoma and every 6 weeks through month 4 for patients with breast cancer, unless alternate systemic therapy was begun for cancer treatment.

Research sample collection included mandatory tumor biopsy at baseline (preinfusion) and within 3 days of last CAR T-cell infusion, if feasible. Peripheral blood was collected at baseline, at each infusion, on day +1 following the first infusion, at day +25, and at each monthly follow-up visit.

### cMET CAR T-cell Manufacturing and Structure

Autologous peripheral blood lymphocytes were obtained via leukapheresis collection at the Apheresis Unit at the HUP, then transferred to the CVPF at the University of Pennsylvania (Philadelphia, PA) for manufacturing. The apheresis product was processed with the CaridianBCT Elutra, a closed system which utilizes counter-flow centrifugal force to separate cell populations based on size and density, to enrich the lymphocyte population. The lymphocytes collected were then washed using the Haemonetics Cell Saver 5 system and resuspended in modified XVIVO-15 media (Lonza). Lymphocytes were stimulated with magnetic Dynabeads conjugated with mouse anti-human CD3 and CD28 (Gibco) and cultured in static tissue culture flasks. On day 5 of culture, cells were transferred to the WAVE Bioreactor. On the final day of the culture (day 9), the magnetic beads were removed (Baxter MaxSep) and the cells were concentrated using the Cell Saver 5. The next day, cells were washed and resuspended in Electroporation Buffer and loaded into the Maxcyte GT Flow Transfection System. Cells were electroporated with the cMet RNA, and allowed to recover for 4 hours and then formulated in infusible cryopreservation media. The cells were not released from the CVPF until FDA-specified release criteria (e.g., cell purity, sterility, potency, pyrogenicity, etc.) were verified.

The final cMET CAR T-cell product thus consists of autologous T cells that have been expanded *ex vivo* using beads conjugated with anti-CD3 and anti-CD28 antibodies. The cells are transfected with anti-cMET mRNA expressing an extracellular scFv targeting cMET fused to an IgG4 hinge and linked to the human CD8α transmembrane domain. The transmembrane domain is also linked to an intracellular bipartite human signaling domain composed of the CD3ζ and 4-1BB costimulatory modules. The human cMET specific scFv is a codon-optimized scFv derived from the 5D5 Genentech antibody.

### Correlative Analyses

Peripheral blood was evaluated for mRNA signals corresponding to cMET CAR T cells using RT-PCR at the following timepoints: preinfusion safety visit; on the day of each infusion before and 1 hour after infusion; at 4 hours following the first infusion; on day +1; on day +25. As described previously (37, 38), for this analysis, cMET CAR T mRNA was measured relative to CD3 mRNA, which served as an endogenous control for T cells. Ratios of mRNA quantities were analyzed as a surrogate for the number of cells expressing cMET CAR.

Formalin-fixed paraffin-embedded tissue sections from preinfusion and postinfusion tumor samples were analyzed by IHC using the Leica Bond III instrument. Slides were pretreated with Bond ER2 solution for 20 minutes at 100°C. Antibodies to CD3 (Leica, PA0122), CD4 (Biocare, API3209AA), CD8 (Dako, M7103), Ki67 (Dako IR626), Foxp3 (BioLegend, 320102), phosphorylated S6 (pS6), PD1 (Abcam, ab52587), PD-L1 (Cell Signaling Technology, 15165BF), CD163 (Leica, CD163-L-CE), granzyme B (Leica, PA0291), and cMET (Ventana, 790-4430) were used as described previously (37, 46). IHC analyses were conducted by a single pathologist (X. Xu) and intensity of staining was designated as low (+), moderate (++), or high (+++).

### Statistical Analyses

The planned sample size for this pilot study was up to 6 evaluable subjects with melanoma and up to 4 evaluable subjects with breast cancer. Subjects were evaluable for all endpoints if they received at least one dose of the RNA CAR T product infusion. If a subject consented but did not undergo cell infusion, that subject could be replaced. Toxicity was tabulated by grade and summarized using descriptive statistics, with patients with melanoma and breast cancer pooled for these analyses. After treatment of every 3 subjects, treatment-limiting events were calculated and an early pausing rule was established such that if significant evidence existed that the event rate of a TLT exceeded the maximum acceptable rate predefined in the protocol, the study should be suspended pending further evaluation. Overall response rate at day +25 (±5 days), progression-free survival (PFS) and overall survival (OS) were also analyzed and reported descriptively. PFS was defined as the time from the date of first infusion to the date of first documented disease progression or death due to any cause. OS was defined as the time from the date of first infusion to the date of death from any cause; if date of death was unavailable, OS was censored at the last date of contact.

### Data Availability

The data generated in this study are available within the article.

## Results

### Subject Characteristics

Between February 2017 and April 2019, 77 patients (38 with TNBC; 39 with melanoma) were consented for prescreening of tumor tissue for quantification of cMET expression. Of all screened subjects, 47 had cMET testing of a metastatic site, 28 had cMET testing on a primary site, and 1 subject had cMET testing performed on both a primary and metastatic site. Median cMET expression was 35% (range: 0%–100%) for the 38 subjects with TNBC and 10% (range: 0%–100%) for the 39 subjects with metastatic melanoma. Ultimately 37 (48%) of 77 prescreened subjects including 20 (53%) patients with TNBC and 17 (44%) patients with melanoma had ≥30% cMET expression by IHC. Of these patients, 4 individuals with TNBC and 3 individuals with melanoma consented and were treated on study.

Characteristics of the 7 subjects enrolled on the therapeutic portion of the study are shown in Table 1. Among 7 subjects treated on study, 1 subject was African American, 5 subjects were White, and race of 1 subject was unknown. Six of 7 subjects were female, 1 was male. Overall, the patient population had received multiple prior lines of chemotherapy and immunotherapy for their cancers. Six of 7 subjects received all planned cell infusions; 1 subject received five infusions due to the quantity of cells available for manufacturing.

**TABLE 1:** Patient population

	TNBC subjects (*n* = 4)	Melanoma subjects (*n* = 3)	All subjects (*n* = 7)
Age (mean, range)	45 (36–58)	49 (35–64)	50 (35–64)
ECOG PS (median, range)	0 (0)	1 (0–1)	0 (0–1)
Sex			
Female	4	2	6
Male	0	1	1
Race			
African American	1	0	1
Caucasian	3	2	5
Unknown	0	1	1
cMET expression (median %, range)	65 (40–100)	50 (30–100)	50 (30–100)
Prior lines of therapy (median, range)			
Chemotherapy	4 (2–4)	1 (0–1)	2 (0–4)
Immunotherapy	0 (0)	3 (2–5)	0 (0–5)

### Adverse Events

All subjects were evaluable for toxicity. Adverse events are shown in Table 2. Six of 7 subjects had at least one adverse event that was possibly related to study therapy. All adverse events were grade 1 or 2 and medically manageable. No grade 3, grade 4, or grade 5 adverse events occurred on study. There were no serious adverse events or TLTs. One subject (Subject 1) experienced cytokine release syndrome (CRS) manifested by low-grade fever (maximum temperature 100.1 degrees Fahrenheit) and arthralgias which resolved within 24 hours without incident. Three clinically relevant adverse events occurred in greater than 1 patient: anemia (*n* = 3), fatigue (*n* = 2), and malaise (*n* = 2).

**TABLE 2:** Adverse events, clinically relevant (# subjects includes those with the given toxicity at least possibly related to study treatment)

Adverse event	Grade 1 (# subjects)	Grade 2 (# subjects)	Grade 3/4 (# subjects)	Total (# subjects)
Anemia	2	1		3
Fatigue	2			2
Malaise	2			2
Arthralgia	1			1
Chest pain	1			1
Cytokine release syndrome	1			1
Dizziness	1			1
Headache	1			1
Nausea	1			1
Vomiting	1			1

No neurotoxicity, anaphylaxis, or allergic reactions were observed. No subjects discontinued study participation due to treatment toxicity.

### Response

Response data are shown in Table 3. Best response on study by RECIST version 1.1 assessed at day +25 was stable disease in 4 patients and disease progression in three; no patients had partial or complete responses. Best response on study by the immune-related RECIST (irRECIST) was stable disease in 3 subjects; progressive disease in 1 subject. Response by irRECIST criteria was not assessed in 3 subjects. No subjects remained on active study until their second planned assessment posttreatment. Median PFS was 0.9 months (range: 0.6–1.7 months). Dates of death were available for 6 subjects; one subject's OS estimate is based on censoring at last follow-up. Median OS was 4.4 months (range: 1.3–24.7 months). There was no correlation between tissue cMET expression at the time of screening and PFS or OS. All subjects discontinued study participation due to disease progression and plan to begin alternate therapy or pursue hospice.

**TABLE 3:** Response

Subject ID	Cancer type	% cells expressing cMET at screening	Best response (RECIST v 1.1)	Best response (irRECIST)	PFS (months)	OS (months)
01	Melanoma	50	SD	Not assessed	1.4	2.9
06	Melanoma	100	PD	irPD	1.0	7.1
27	TNBC	40	SD	irSD	1.7	11.5
31	TNBC	80	SD	irSD	0.9	24.7
56	TNBC	50	PD	Not assessed	0.6	1.3
67	TNBC	100	PD	Not assessed	0.9	4.4
74	Melanoma	30	SD	irSD	0.9	2.1

Abbreviations: irPD: immune related response progressive disease and irSD: immune related response stable disease.

### cMET CAR T mRNA detection by RT-PCR

mRNA signals corresponding to CAR T cells were detected by RT-PCR in the peripheral blood of all patients on all days that they received infusions, 1 hour following infusions. Table 4 shows the ratio of cMET CAR signals to CD3 signals × 100. On day 0, mRNA signals corresponding to CAR T cells declined in all patients from 1 to 4 hours after infusion. Day +1 (the day in between the first and second infusions) mRNA signals corresponding to CAR T cells were detected in the peripheral blood of 3 subjects. On all infusion days, an increase in peripheral blood mRNA signals corresponding to CAR was seen following infusion. Four subjects had evaluable peripheral blood samples from day +25 visits, and mRNA signals corresponding to CAR T cells were not detected in any of these samples. No peripheral blood was collected for any subject after day +25 to evaluate for persistence. Postinfusion tumor tissue from 5 subjects was evaluated for mRNA signals corresponding to CAR T cells, with no evidence of cMET CAR found.

**TABLE 4:** mRNA signals corresponding to CAR T cells in peripheral blood

	Day 0, Infusion #1				Day 2		Day 4		Day 7		Day 9		Day 11		
Subject ID	Pre	1 hour Post	4 hours Post	Day 1	Pre	1 hour Post	Pre	1 hour Post	Pre	1 hour Post	Pre	1 hour Post	Pre	1 hour Post	Day 25
**1**	ND	0.06	0.006	ND	ND	0.03	ND	0.12	ND	0.04	ND	0.08	ND	0.04	ND
**6**	ND	0.015	0.002	ND	ND	0.029	ND	0.243	ND	0.333	ND	0.007	ND	0.003	ND
**27**	ND	0.03	0.002	0.0007	0.0009	0.02	0.002	0.05	0.002	0.03	0.002	0.02	0.002	0.06	ND
**31**	ND	0.083	0.012	0.012	ND	0.018	ND	0.314	ND	0.181	0.002	0.062	0.001	0.068	NA
**56**	ND	0.03	ND	ND	0.003	0.02	ND	0.04	0.002	0.02	0.002	0.08	NA		NA
**67**	ND	0.03	0.007	0.003	0.001	0.46	ND	0.02	0.001	0.03	0.004	0.02	ND	0.09	NA
**74**	ND	0.06	0.04	ND	0.001	0.14	ND	0.12	0.005	1.7	0.003	0.024	ND	0.01	ND

NOTE: Values shown indicate cMET CAR mRNA/CD3 mRNA × 100.

Abbreviations: ND, cMET CAR mRNA not detected; NA, sample not collected and not available for analysis.

### IHC Analysis of Paired Tumor Tissue

**FIGURE 2:**
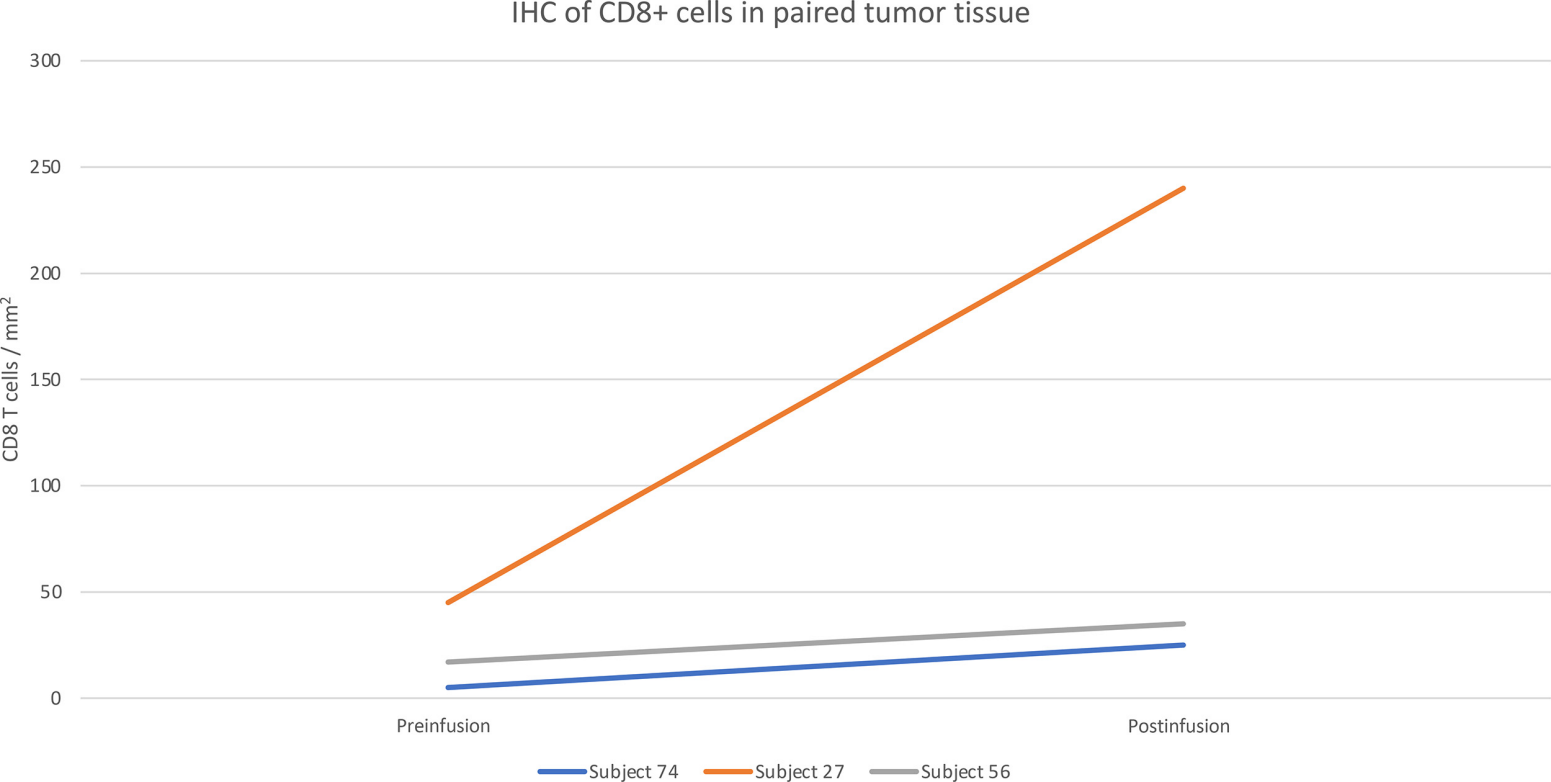
IHC analysis of CD8 in preinfusion and postinfusion tumor tissue. Three subjects had preinfusion and postinfusion tumor tissue available for analysis; postinfusion tumor tissue demonstrated an increase in cytoxic CD8^+^ cells in all 3 subjects.

Three subjects had preinfusion and postinfusion tumor tissue analyzed for markers of immune activation (CD3, CD4, CD8), regulatory T cell function (Foxp3) and tumor cell proliferation (Ki67, pS6; Table 5). Decreases in pS6 were noted in 2 subjects, and 1 subject had a decrease in Ki67 (Supplementary Fig. S1). All 3 subjects had an increase in cytotoxic CD8^+^ cells noted (Fig. 2; Supplementary Fig. S2), and 2 subjects also had increases in CD3^+^ cells. There was no change in PD1, PDL1, or cMET expression postinfusion in subjects for whom paired tissue was available.

**TABLE 5:** IHC analysis of paired tumor tissue

	Subject 74		Subject 27		Subject 56	
	Preinfusion	Postinfusion	Preinfusion	Postinfusion	Preinfusion	Postinfusion
CD3	+	++	+	++	+	+
CD4	+	+	+	++	+	+
CD8	+	++	+	++	0	+
Ki67	++	+	++	++	+++	+++
Foxp3	+	++	0	+	+	+
pS6	+++	+	+++	+++	+++	+
PD1	0	0	Not performed	+	+	+
PDL1	0	0	Not performed	+	+	+
CD163	++	++	Not performed	++	+	++
GranzymeB	0	0	Not performed	+	0	0
cMET	++	++	++	++	++	++

## Discussion

CAR T-cell therapy can confer effective and durable disease control for patients with B-cell hematologic malignancies. To date, however, CAR T efforts have been less successful in solid tumors due to restricted trafficking to, infiltration of, and activation within tumors, in the context of potentially immunosuppressive microenvironments. Additional challenges include on-target, off-tumor effects due to the presence of tumor-associated antigens in physiologically present tissues. Here, we present a pilot clinical trial of RNA-electroporated T cells expressing a CAR directed against the cMET protein in patients with metastatic melanoma and TNBC. To our knowledge, the current study represents the first published clinical trial of systemic CAR T administration in patients with melanoma and breast cancer.

The rationale for selecting this construct was based on the following: first, the cMET antigen was felt to have substantial expression on melanoma and TNBC cells with somewhat limited expression on physiologic tissues; second, an RNA vector was felt to have a lower risk of severe and persistent on-target, off-tumor toxicities as compared with a lentiviral vector, and third, limited available data suggested that the mRNA approach could potentially yield antitumor activity. A prior study of intratumoral cMET CAR T injections demonstrated tumor necrosis and an inflammatory response at the site of therapy without substantial toxicity, specifically hepatotoxicity (37). In addition, prior studies examining RNA-electroporated mesothelin-directed CAR T cells in patients with malignant pleural mesothelioma and pancreas cancer demonstrated the potential of systemically infused mRNA CAR T cells to traffick into the tumor microenvironment and induce disease stability and clinical response (38, 39). These data supported the investigation of the mRNA cMET CAR T construct in the current study, which aimed to evaluate the safety of systemic administration.

Seventy-seven subjects with breast cancer and melanoma consented to tumor prescreening for potential involvement in this CAR T study, demonstrating patients’ interest in, and providers’ support for exploring CAR T approaches, a necessary element for feasibility of CAR T trials in solid tumors. In the current study, the eligibility threshold for cMET expression was 30%, and approximately half of subjects screened met this threshold. The median cMET expression for patients with mTNBC was 35% and the median cMET expression for patients with melanoma was 10%. This is lower than target expression of CAR T therapy targets for hematologic indications; for B-cell hematologic malignancies, a minimum CAR T target antigen expression is not specified since substantial expression is nearly universal. It is possible that either cMET is not an optimal target for CAR T approaches in metastatic melanoma and TNBC, or that the target expression threshold for CAR T approaches in solid tumors may be lower than in leukemia and lymphoma.

This pilot study involved systemic administration of RNA-electroporated cMET CAR T without lymphodepleting chemotherapy in six planned infusions over an elapsed 2 weeks. Dose escalation, for example with doses greater than 1 × 10e8 cells or including cohorts with lymphodepleting chemotherapy were not planned as part of this study, given the pilot nature of this trial and the original plan for a lentiviral study that might include such features. This schedule was selected in light of the short-lived RNA construct and it proved feasible, with only 1 patient having five of six planned infusions due to the quantity of manufactured cells. Treatment was also safe, with only grade 1 and grade 2 adverse events that were medically manageable, and no serious adverse events or TLTs. One patient experienced grade 1 CRS, manifested only by low-grade fever that resolved without intervention. No neurotoxicity occurred on study. Anaphylaxis felt to be mediated by the development of human anti-CAR IgE antibodies has been noted after repeated, intermittent mRNA CAR T-cell infusions (43) and importantly, was not seen in this study. Physiologic expression of cMET is seen in the liver, pancreas, prostate, kidney, muscle, and bone marrow. Aside from anemia (grade 1 in 2 subjects; grade 2 in 1 subject), no target organ-specific toxicities were seen. No subjects discontinued study participation due to treatment toxicity.

In this heavily pretreated population, no responses were seen by either RECIST or irRECIST. PFS and OS were low, with a median PFS of 0.9 months (range: 0.6–1.7 months) and median OS of 4.4 months (range: 1.3–24.7 months). Clinical activity was not seen. It is possible that the lack of treatment response was related to limited trafficking to tumor, given that postinfusion tumor tissue from 5 subjects demonstrated no evidence of cMET CAR. In addition, mRNA CAR T cells have a limited quantity of RNA and there is a dilution of CAR expression upon proliferation, potentially limiting clinical activity. The absence of lymphodepletion may have contributed to lack of efficacy. In a study of patients with Hodgkin lymphoma treated with CD19-directed RNA-electroporated CAR T cells, transient responses were seen; lymphodepleting chemotherapy was administered prior to cell infusion (47). Finally, novel approaches may be required to overcome the immunosuppressive solid tumor microenvironment. Preclinical evaluation of cMET-directed adoptive immunotherapy includes bispecific and chimeric switch receptor approaches in hepatocellular carcinoma (48–50), gastric cancer (51, 52), renal cell carcinoma (53), and non–small cell lung cancer (54). These efforts include cMET CAR-NK (natural killer) cell immunotherapy (50), bispecific CAR constructs targeting both cMET and PDL1 (48, 55) as well as cMET CAR T approaches utilizing a PD1/CD28 chimeric-switch receptor aimed at promoting antitumor activity by reversing PD1-mediated immunosuppression (51). A clinical trial has been designed to evaluate the efficacy and safety of cMET/PDL1 CAR T-cell therapy in patients with hepatocellular carcinoma; however, the status of this trial is not known (56). Notably however, the subject who experienced grade 1 CRS (Subject 1) had 50% expression of cMET at screening and achieved stable disease on study with a PFS of 1.4 months, which is longer than most other subjects on study.

mRNA signals corresponding to CAR T cells were detected in the peripheral blood of 3 unique subjects on the day following the first infusion and in some patients, on the days of infusion, prior to infusion. However, no such signals were seen in day +25 samples or in available postinfusion tumor tissue. In contrast to CAR engineering using viral vector transduction resulting in genomic integration, CAR expression through RNA electroporation allows for a transiently expressed CAR molecule on the surface of delivered T cells (57). The absence of mRNA signals corresponding to CAR T cells at day +25 or in postinfusion tumor tissue indicates limited persistence, consistent with the current understanding of RNA-based CAR T constructs. It is possible that the lack of responses was a result of the limited expansion, trafficking, and infiltration of CAR T cells to sites of disease, and that clinical activity may have been seen if a more durable construct was used. It is also possible that the lack of responses was related to the magnitude of target antigen expression. Although first response evaluation was at day +25 after first infusion, pseudoprogression and delayed responses have not been a prominent feature of CAR T-cell therapy response and it is therefore unlikely that a later imaging timepoint would have captured more responses.

IHC analysis was performed on 3 subjects who had available paired [preinfusion and postinfusion on day 11 or within 3 days of last CAR T infusion (Table 5)]. The increases seen in CD8^+^ and CD3^+^ cells are concordant with an increase in immune activation, and the decreases in Ki67 and pS6 may indicate a decrease in tumor cell proliferation. Despite these IHC indicators of CAR T-cell activity in Subjects 56 and 74, mRNA signals corresponding to cMET CAR was not detected in postinfusion tumor tissue. This is possibly due to timing of biopsy in the context of the short-lived mRNA constructs or due to low migration and penetration of tumor tissue by CAR T cells, noting that lack of antecedent lymphodepleting chemotherapy may have limited CAR T-cell expansion and survival. However, conclusions cannot be drawn due to the small number of patients.

Consideration was given to expansion of the current clinical trial with additional subjects treated with RNA-electroporated cMET CAR T cells or evaluation of lentiviral cMET CAR T-cell therapy in patients with metastatic TNBC and/or metastatic melanoma. Given the therapeutic landscape in both disease subtypes which broadened over the study period to include additional treatments including checkpoint inhibition, the decision was made to no longer pursue the RNA-electroporated cMET CAR T approach in metastatic TNBC or melanoma. Evaluation using a lentiviral construct may yield improved efficacy. A higher cMET expression threshold may also help to maximize efficacy.

This pilot study of RNA electroporated cMET CAR T cells evidences safety of RNA-electroporated cMET CAR T cells and feasibility of the CAR T-cell approach in subjects with advanced solid tumors, and given the promise of CAR T approaches in relapsed liquid malignancies, further study of CAR T-cell therapy in patients with advanced solid tumors is warranted. Thoughtfully selected, highly-expressed, tumor-specific antigens and use of a durable construct may maximize efficacy.

## Supplementary Material

Supplementary Figure 1 Supplementary Figure 1.: Representative immunohistochemical staining for Subject 74 with metastatic melanoma; lymph node biopsy. Panel A: pS6 staining prior to infusion; Panel B: pS6 staining at post-infusion biopsy; Panel C: Ki-67 staining prior to infusion; Panel D: Ki-67 staining at post-infusion biopsy.

Supplementary Figure 2 Supplementary Figure 2: Representative immunohistochemical staining for Subject 27 with metastatic triple-negative breast cancer; lymph node biopsy. Panel A shows CD8 staining prior to infusion and Panel B shows CD8 staining at the time of post-infusion biopsy.
