# Supplementary figures and images for: Phase I Trial of Autologous RNA-electroporated cMET-directed CAR T Cells Administered Intravenously in Patients with Melanoma and Breast Carcinoma
Source: Cancer Res Commun. 2023 May 9;3(5):821–9. doi: 10.1158/2767-9764.CRC-22-0486 (PMC10167933; doi:10.1158/2767-9764.CRC-22-0486)

## Slide 1
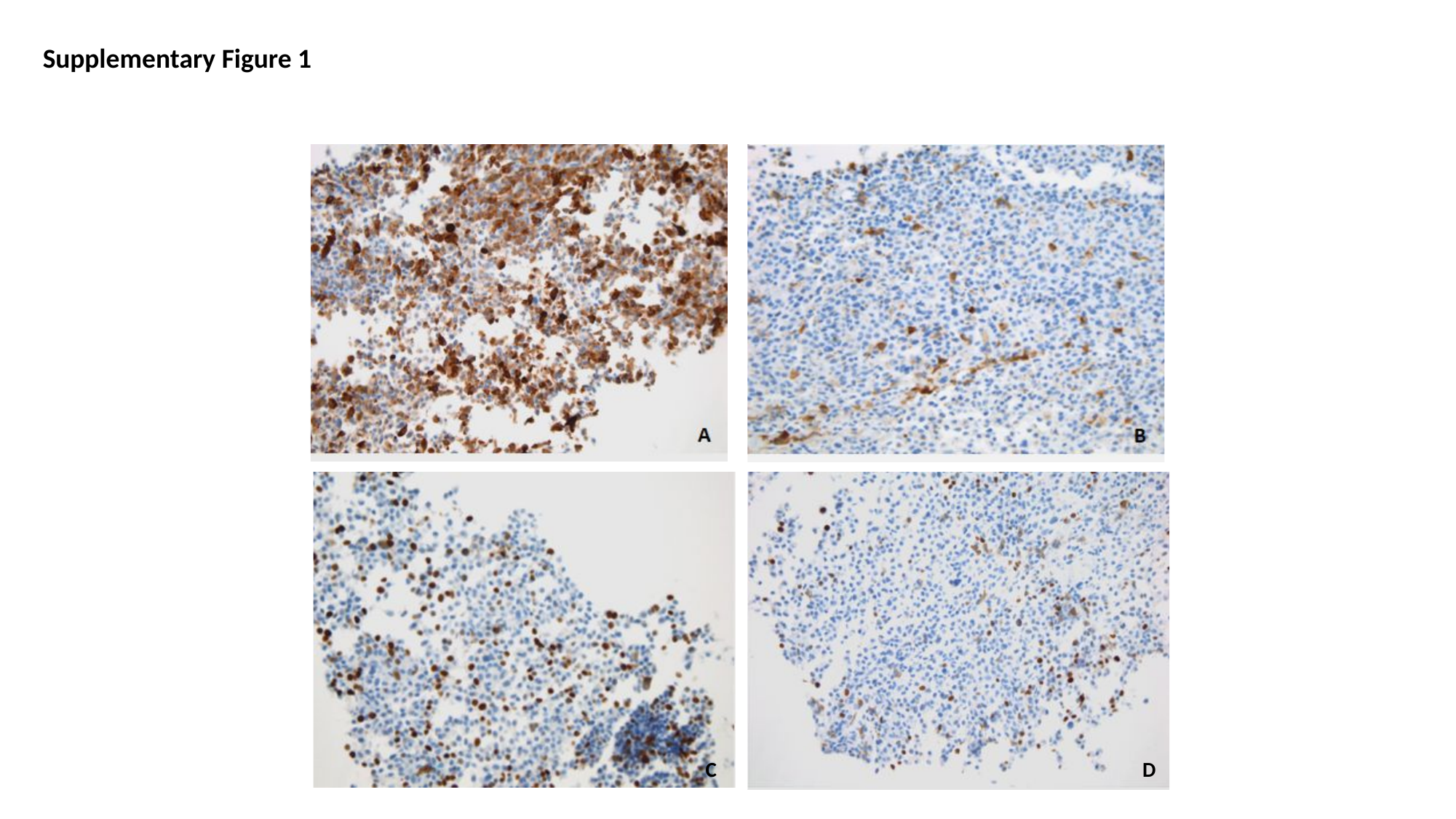

Supplementary Figure 1
C
D

Supplement: Supplementary Figure 1 — Representative immunohistochemical staining for Subject 74 with metastatic melanoma; lymph node biopsy. Panel A: pS6 staining prior to infusion; Panel B: pS6 staining at post-infusion biopsy; Panel C: Ki-67 staining prior to infusion; Panel D: Ki-67 staining at post-infusion biopsy. [file crc-22-0486-s01.pptx]

## Slide 1
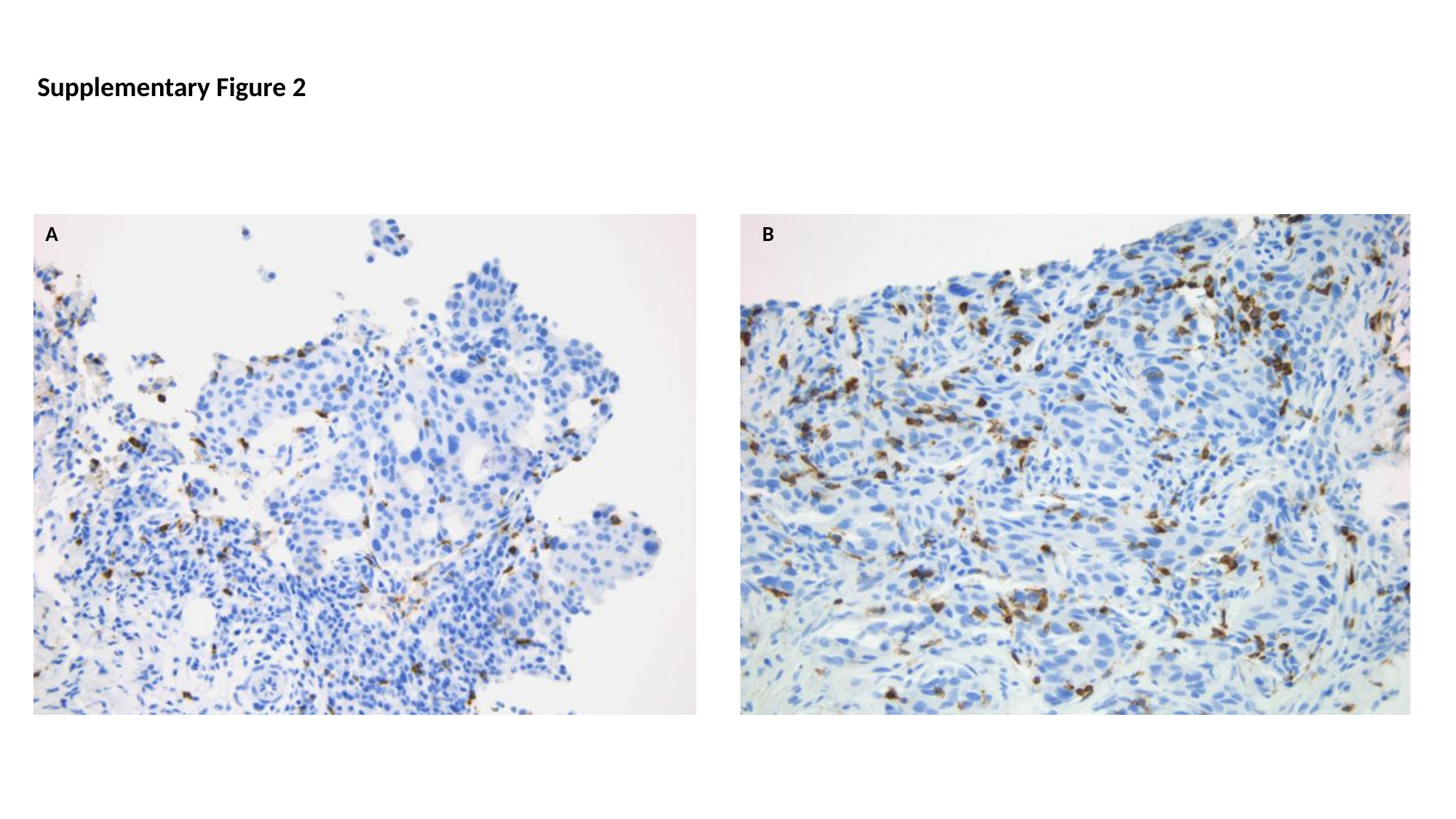

Supplementary Figure 2
A
B

Supplement: Supplementary Figure 2 — Representative immunohistochemical staining for Subject 27 with metastatic triple-negative breast cancer; lymph node biopsy. Panel A shows CD8 staining prior to infusion and Panel B shows CD8 staining at the time of post-infusion biopsy. [file crc-22-0486-s02.pptx]
